# *Trichomonas vaginalis and Giardia lamblia* Growth Alterations by Low-Frequency Electromagnetic Fields

**Published:** 2019

**Authors:** Abraham Octavio RODRÍGUEZ-DE LA FUENTE, Ricardo GOMEZ-FLORES, José Antonio HEREDIA-ROJAS, Edna Marbella GARCÍA-MUÑOZ, Javier VARGASVILLARREAL, Magda Elizabeth HERNÁNDEZ-GARCÍA, Francisco GONZÁLEZSALAZAR, Jesús Norberto GARZA-GONZÁLEZ, Michaela BELTCHEVA, Omar HEREDIA-RODRÍGUEZ

**Affiliations:** 1. Department of Exact Sciences and Human Development, Biological Sciences School, Autonomous University of Nuevo León, Sán Nicolás de los Garza, Nuevo León, México; 2. Department of Immunology and Virology, Biological Sciences School, Autonomous University of Nuevo León, Sán Nicolás de los Garza, Nuevo León, México; 3. Northeast Biomedical Research Center, Mexican Institute of Social Security, Monterrey, Nuevo León, México; 4. Institute of Biodiversity and Ecosystem Research, Bulgarian Academy of Sciences, Sofia, Bulgaria

**Keywords:** *Trichomonas vaginalis*, *Giardia lamblia*, Magnetic fields, Parasite growth

## Abstract

**Background:**

There is an increasing interest in using physical factors such as magnetic fields as antimicrobial strategy, with variable results. The current study was aimed to evaluate the influence of extremely low-frequency electromagnetic fields (ELF-EMFs) on the axenically-cultured parasite protozoans *Trichomonas vaginalis* and *Giardia lamblia* growth.

**Methods:**

Bioassays were developed using *T. vaginalis*, GT-13 and *G. lamblia* IMSS-0989 strains cultured at 37 ºC in TYI-S-33 medium. The following treatment regimens and controls were considered: (a) cells exposed to ELF-EMFs, (b) untreated cells, (c) cells treated with Metronidazole, used as positive controls, and (d) cells co-exposed to ELF-EMFs and Metronidazole. When cultures reached the end of logarithmic phase, they were exposed to ELF-EMFs for 72 h, in a standardized magnetic field exposure facility. For determining cytotoxic effects, trophozoite density was blindly evaluated in a Neubauer chamber.

**Results:**

A significant decrease in trophozoite growth was observed for *T. vaginalis*, in magnetic field-treated cultures. On the other hand, cultures co-exposed to ELF-EMFs and Metronidazole showed no significant differences when compared with cultures treated with Metronidazole alone. On the contrary, an increased trophozoite density was observed in *G. lamblia* cultures after exposure to magnetic fields. An absence of a synergistic or antagonistic effect was observed.

**Conclusion:**

ELF-EMFs induced *T. vaginalis* and *G*. *lamblia* growth alterations, indicating a potential effect in cell cycle progression.

## Introduction

There is a trend towards the use of magnetic fields in antimicrobial therapy, with variable results. Currently, the use of antibiotics has been the choice to control parasite infections, however side effects constitutes a major concern. In contrast, physical factors have become an effective alternative to drugs.

Regarding parasite growth inhibition using physical procedures, in our laboratory it has been demonstrated in vitro inhibition of *Entamoeba invadens,* and *Entamoeba histolytica* and *Trichomonas vaginalis* growth by magnetic fields and bioresonance, respectively (1,2).

To date, there are no reports of the effect of extremely low-frequency electromagnetic fields (ELF-EMFs) on *T. vaginalis* and *G. lamblia* growth. However, studies have been conducted in protozoans. In this regard, Amaroli et al (3) observed a decreased *Dictyostelium discoideum* growth after exposure to ELFEMFs. Additionally, *Entamoeba histolytica* and *E. dispar* growth inhibition was observed after 900 MHz electromagnetic fields treatment (4).

In view of this compelling issue involving magnetic field effects on parasites, the present study was undertaken to evaluate ELF-EMFs on *T. vaginalis* and *G. lamblia* growth.

## Materials and Methods

### Parasites

The current study was developed using *T. vaginalis* GT-13 and *G. lamblia* IMSS-0989 strains cultured at 37ºC in TYI-S-33 medium, as previously described (5).

### Experimental design

The following experimental groups (27 cultures per group) were used: (a) cells exposed to ELF-EMFs at 60 Hz and 2.0 mT, (b) untreated cells, (c) cells treated with Metronidazole (a well-known anti-parasite drug) at 0.14 μg/ml for *T. vaginalis* and 0.56 μg/ml for *G. lamblia*, used as positive controls, and (d) cells co-exposed to ELF-EMFs and Metronidazole, at the same concentration of 0.14 μg/ml for *T. vaginalis* and 0.56 μg/ml for *G. lamblia*.

### Electromagnetic field exposure facilities and measurements

When cultures reached the end of logarithmic phase, they were exposed to a 60 Hz and 2.0 mT ELF-EMF for 72 h. A standardized magnetic field exposure facility of identical characteristics as one used in our previous work (6) was used. This device comprised a coil that was built by winding 340 turns of 1.3 mm diameter enamel insulated copper wire to form a cylindrical solenoid with a radius of 5.27 cm and a length of 25 cm. The solenoid was connected to a step-down transformer and to a variable transformer that was plugged in to a 110 V AC source.

Magnetic flux density (rms) was measured using an axial Hall-effect probe (Bell FW 6010 gaussmeter, Orlando, Fl, USA). An oscilloscope (BK-Precision model 2120) was coupled to the system to monitor the resultant field. A 2.0 mT (rms) 60 Hz alternating sinusoidal electromagnetic field was then generated. The electromagnetic field frequency content was nearly pure 60 Hz (<3% total harmonic distortion). To keep the geometry of exposure, a plastic separator was placed inside the solenoid to allow the placement of cultures in predetermined zones where the rms value of the ELF-EMF was homogeneous. The magnetic field ambient background level was < 0.8μT. Moreover, the local geomagnetic field was also measured, setting the gaussmeter in DC mode and by using an axial high sensitivity Hall probe (Integrity Design IDR-321 geo-magnetometer, Essex Jct., VT). The average value was 20 mT within the exposure room.

### Co-exposure to magnetic fields and Metronidazole

In order to test the combination of 60 Hz ELF-EMF treatment and Metronidazole (Sigma-Aldrich, St. Louis, MO, USA) on parasite growth, a co-exposure experiment was included. Metronidazole was dissolved in TYIS-33 medium and adjusted to the above cited concentrations. Cultures were co-exposed at the same time for 72 h.

### Bioassays

For determining cytotoxicity, trophozoite density was blindly evaluated in triplicate by using a hemocytometer Neubauer chamber.

### Statistical analysis

The statistical differences were calculated among groups by using an analysis of variance for normal distributions and the correspondent parametric Turkey test for establishing individual differences. The normality of the data was estimated by means of KolmogorovSmirnov test. All analyses were performed using the SPSS package version 22.0 (Chicago, IL, USA). Differences were considered to be significant when the *P* values were lower than 0.05. Moreover, the exact *P* values for individual differences are given in the caption of the figures of results.

## Results

**Fig. 1:**
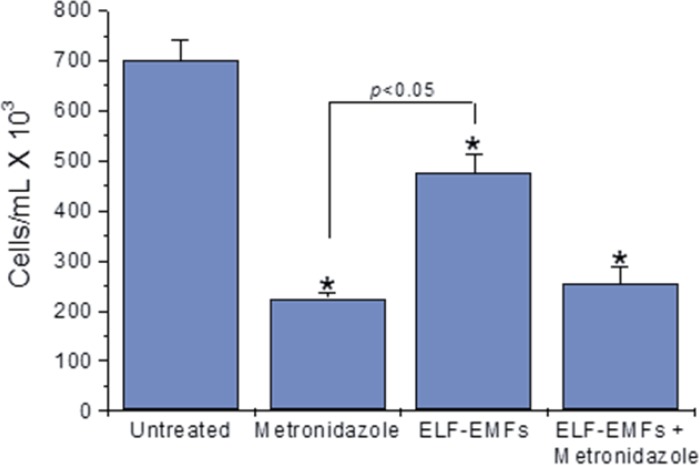
Effect of 60 Hz sinusoidal magnetic fields at 2.0 mT and 72 h exposure on *T. vaginalis* trophozoite growth. Bars represent arithmetical grouped means ± standard error. The *P* values for individual differences are: Untreated cells vs Metronidazole treatment (4.98 × 10^–13^), untreated cells vs ELF-EMF exposure (3.00 × 10^–6^), untreated cells vs co-exposure condition (4.98 × 10^–13^)

In magnetic field-treated cultures, a significant *T. vaginalis* trophozoite growth inhibition was observed (Fig. 1). In addition, cultures co-exposed to ELF-EMFs and metronidazole showed no significant differences, when compared with cultures treated with metronidazole alone. However, cultures treated with metronidazole showed lower cell density as compared with negative controls, as expected.

**Fig. 2:**
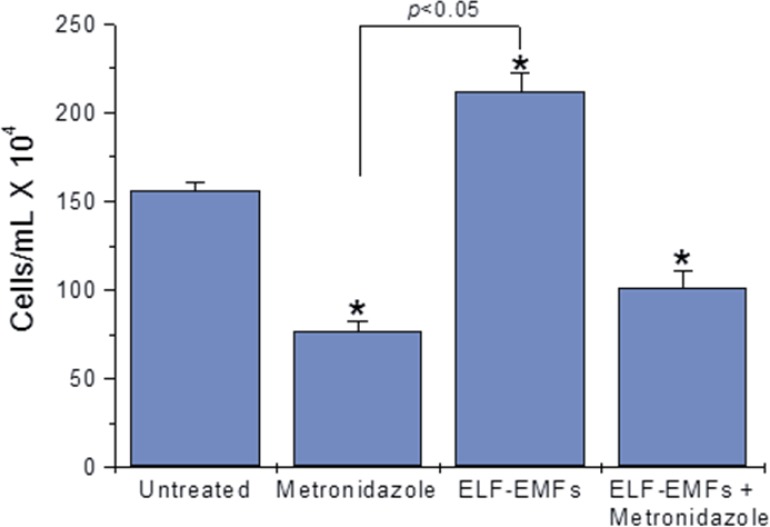
Effect of 60 Hz sinusoidal magnetic fields at 2.0 mT and 72 h exposure on *G. lamblia* trophozoite growth. Bars represent arithmetical grouped means ± standard error. The *P* values for individual differences are: Untreated cells vs Metronidazole treatment (7.51 × 10^–12^), untreated cells vs ELF-EMF exposure (3.90 × 10^–7^), untreated cells vs co-exposure condition (5.97 × 10^–7^)

In contrast, ELF-EMFs induced *G. lamblia* increased growth, and no effect of combination with metronidazole was observed, indicating the absence of a synergistic or antagonistic effect (Fig. 2).

## Discussion

In the current study, we have observed growth alterations in parasite protozoans *T. vaginalis* and *G. lamblia* after axenic cultures were exposed to ELF-EMFs. In regard to *T. vaginalis*, the observed results agreed with our previous report on *E. invadens* growth inhibition after 2.0 mT exposure (1).

In *G. lamblia* experiments, an increased trophozoite density was shown after exposure to ELF-EMFs. In a previous study, we found an increased human lymphocytes proliferation when exposed to 2.0 mT magnetic fields (6). For instance, Dihel et al (7) observed an increased cell proliferation in cultures of *Paramecium tetraurelia* exposed to ELF-EMFs.

On the other hand, our findings suggested that lower cell counts in *T. vaginalis* were related to ELF-EMF exposure, which may involve alterations in cell cycle progression. It has been suggested by others that electroporation induced by magnetic fields destroys cell membranes and acts as an antimicrobial procedure (8). Another possibility is that the parasites reacted to magnetic fields in a similar way to that observed under cellular stress. This idea is supported by our observation that there is an increase in heat shock gene transcripts (e.g., hsp70) following exposure to ELF-EMFs (9).

Our results showed no synergistic or antagonistic effects of ELF-EMFs and Metronidazole combination. We previously observed an antagonistic effect between magnetic fields and Mitomycin-C on human lymphocytes proliferation (6), and for chromosomal aberrations and sperm morphology in germ cells of mice (10).

## Conclusion

ELF-EMFs induced *T. vaginalis* and *G. lamblia* growth alterations. However, with the results presented here, we are not supporting nor recommending electromagnetic approaches, rather we showed evidence for a significant and measurable biological effect induced by magnetic fields on parasite protozoans.
